# Bridging the gap: functional healing of embryonic small intestine *ex vivo*

**DOI:** 10.1002/term.2073

**Published:** 2015-08-03

**Authors:** Riccardo Coletta, Neil A. Roberts, Francesca Oltrabella, Basem A. Khalil, Antonino Morabito, Adrian S. Woolf

**Affiliations:** ^1^ Institute of Human Development, Faculty of Medical and Human SciencesUniversity of ManchesterUK; ^2^ Paediatric Autologous Bowel Reconstruction and Rehabilitation Unit, Department of Paediatric SurgeryRoyal Manchester Children's HospitalUK; ^3^ Faculty of Life SciencesUniversity of ManchesterUK; ^4^ Institute of Inflammation and Repair, Faculty of Medical and Human SciencesUniversity of ManchesterUK

**Keywords:** E‐cadherin, embryo, jejunum, lumen, organ culture, peripherin, peristalisis, smooth muscle

## Abstract

The ability to grow embryonic organs *ex vivo* provides an opportunity to follow their differentiation in a controlled environment, with resulting insights into normal development. Additionally, similar strategies can be used to assess effects on organogenesis of physical and chemical manipulations. This study aimed to create an organ culture model with which to test physical manipulations to enhance healing of gut segments, thus generating a single functional organ. Embryonic mouse jejunum was isolated and cut into 2–3 mm tubes, which were placed in pairs, separated by a small gap, on semi‐permeable supports. Each pair was linked by a nylon suture threaded through their lumens. After 3 days in organ culture fed by defined serum‐free media, the rudiments differentiated to form tubes of smooth muscle surrounding a core of rudimentary villi. Of 34 such pairs, 74% had touching and well aligned proximate ends. Of these joined structures, 80% (59% of the total pairs) had a continuous lumen, as assessed by observing the trajectories of fluorescent dextrans injected into their distal ends. Fused organ pairs formed a single functional unit, as assessed by spontaneous contraction waves propagated along their lengths. In these healed intestines, peripherin^+^ neurons formed a nexus in the zone of fusion, linking the rudiment pairs. In future, this system could be used to test whether growth factors enhance fusion. Such results should in turn inform the design of novel treatments for short bowel syndrome, a potentially fatal condition with a currently limited and imperfect range of therapies. ©2015. The Authors Journal of Tissue Engineering and Regenerative Medicine Published by John Wiley & Sons, Ltd

1

The ability to grow embryonic organs *ex vivo* provides opportunities to follow their differentiation in a controlled environment, with resulting insights into normal development. In addition, *ex vivo* organ culture can be used to assess the effects on organogenesis of physical manipulations or the addition of exogenous chemicals, such as growth factors. With respect to the mammalian small intestine, previous studies have begun to explore these aspects using, as examples, organ culture of intact embryonic gut rudiments (Abud *et al*., 2005; Quinlan *et al*., 2006) and the generation of gut organoids derived from intestinal stem cells (Sato and Clevers, 2013). Our study aimed to create an organ culture model with which to test physical manipulations to enhance healing of small intestinal rudiments, thus generating a single functional organ.

Animal experiments were approved by the Registered Medical and Scientific Departments of the University of Manchester. Wild‐type CD1 mice were bred in the Biological Services Facility and were time‐mated, with the morning of the vaginal plug designated embryonic day 0. After Schedule 1 humane killing, embryonic day 14 proximal small bowel, the forming jejunum, was isolated and cut into 2–3 mm segments. For serum‐free organ culture, a protocol was used which has been shown to support the differentiation of epithelial and mesenchymal/smooth muscle lineages in embryonic mouse bladders (Burgu *et al*., 2006), ureters (Tai *et al*., 2013) and metanephric kidneys (Anders *et al*., 2013). Rudiments were placed onto semipermeable Millicell culture plate inserts made from polytetrafluoroethylene with 0.4 µm pores (Millipore) and maintained at 37°C in a humidified atmosphere of air/5% CO_2_. These platforms provide a solid base on which organs can differentiate and grow, while accessing chemical nutrients from medium below the platform. Explants were fed defined serum‐free medium, placed underneath and touching the platform. ‘Basal medium’ comprised Dulbecco's modified Eagle's medium (DMEM)/F12 (Gibco BRL), insulin (10 mg/l), sodium selenite (5 mg/l) and transferrin (5.5 mg/l). For some experiments, this medium was supplemented with R‐spondin 1 (100 µg/l; Peprotech), a factor that promotes canonical WNT/*β*‐catenin signalling and stimulates intestinal epithelial proliferation in postnatal mice (Kim *et al*., 2005). The explants were observed daily using an inverted microscope (Leica) until day 3, the limit of the study. For some experiments, a sterile polyamide (Nylon) 10/0 suture (Ethicon) was threaded through the lumens of two adjacent rudiments. To determine whether these lumens became continuous during culture, fluorescent fixable dextrans, 2 g/l in phosphate‐buffered saline (PBS; Molecular Probes), were injected into opposite ends of adjacent explants, using a MPPI‐2 Pressure Injector/BP15 Back Pressure Unit (Applied Scientific Instrumentation). Green fluorescent AlexaFluor 488 dextran (10 kDa) was injected into one end and Texas red (70 kDa) into the other. In addition, 2 mm segments of embryonic day 17 jejunum were placed into organ culture (basal medium only) and, after 1 h, they were observed to determine whether they underwent peristalsis.

For whole‐mount immunofluorescence, rudiments were washed in 1% bovine serum albumin (BSA) in 1% PBS and 0.1% Triton X‐100 for 2 h, then blocked with 1% BSA in 1% PBS, 0.1% Triton X‐100 and 5% hea*t*‐treated goat serum for one h. After rinsing, they were incubated for 72 h with 1:500 dilutions of primary antibodies to E‐cadherin (76055, Abcam), an epithelial cell–cell adhesion protein (Tai *et al*., 2013), and peripherin (1530, Millipore), an enteric neuron cytoskeletal protein (Ganns *et al*., 2006). After rinsing, the specimens were incubated overnight with corresponding secondary antibodies. For histology, the guts were cryosectioned at 10 µm and the sections were fixed in acetone for 5 min, washed with ice‐cold 1% PBS and permeabilized in 1% PBS and 0.1% Triton X‐100 for 10 min. After blocking in 1% fish gelatin in 1% PBS and 0.1% Triton X‐100 for 1 h, the sections were incubated for 30 min with goat anti‐*α*‐smooth muscle actin (*α*SMA; Sigma, SAB2500963), a visceral muscle cytoskeletal protein (Wilm *et al*. 2005), and the mouse anti E‐cadherin antibody described above. Endogenous mouse immunoglobulins were blocked using the Mouse on Mouse Kit (Vector Laboratories, BMK‐2202). After washing, appropriate secondary antibodies were applied for 1 h. Finally, 4′,6‐diamidino‐2‐phenylindole (DAPI) staining was undertaken to detect cell nuclei. Dextrans were visualized through an Olympus BX51 microscope and images captured with a Coolsnap ES camera (Photometrics) through MetaVue Software (Molecular Devices). Immunofluorescence was visualized with a Fluoview FV1000 confocal microscope (Olympus) and images were acquired using Fluoview v. 3.1b software (Olympus). The images were then processed and analysed using ImageJ (http://rsb.info.nih.gov/ij) and Adobe Photoshop CS5. Specific band pass filter sets were used to prevent bleed‐through between channels.

As described (Wilm *et al*., 2005), we found that embryonic day 14 proximal small bowel contained a simple epithelium, without villi, surrounded by a mesenchymal layer, the outermost cells of which were *α*SMA^+^ (see supporting information, Figure S1), showing that they had begun differentiation into visceral muscle. After 3 days in culture, rudiments grown in basal medium had elongated and the histology showed intact tissue with the formation of rudimentary villi (see supporting information, Figure S1). The level of tissue organization of embryonic day 14 explants cultured for 3 days was similar to that observed in the freshly‐dissected embryonic day 17 jejunum (see supporting information, Figure S1). In all three conditions (i.e. freshly dissected embryonic day 14 and 17 intestines, and the former cultured for 3 days), the epithelia immunostained for E‐cadherin (see supporting information, Figure S1). Preliminary experiments (data not shown) demonstrated that extended culture periods, for up to 1 week, were incompatible with tissue viability, probably because the organs had become too large to be optimally sustained by the culture system. Therefore, all further experiments used explants grown for 3 days.

We sought to determine whether two explants, comprising adjacent segments *in vivo*, placed near each other in the same proximal–distal orientation as found *in vivo*, might fuse in culture. However, preliminary experiments (data not shown) demonstrated that the rudiments curved as they grew, with the presumed mesenteric side in the concavity, so that the proximate ends of adjacent explants became poorly aligned. Therefore, in an attempt to maintain orientation, upon being explanted the rudiment pairs were linked with a single suture threaded through their lumens (Figure 1A). Using this strategy, as assessed by observations through an inverted microscope, the explants grew as linear tubes. On day 3 of culture, 25 (74%) of 34 such rudiment pairs were noted to be touching, with well‐aligned central epithelial zones (Figure 1B). In 80% of such well‐aligned pairs (i.e. equivalent to 59% of the total explanted pairs), a single patent lumen was confirmed by visualizing the trajectories of injected fluorescent dextrans which crossed the midline of the fused rudiments (Figure 1C–E).

**Figure 1:**
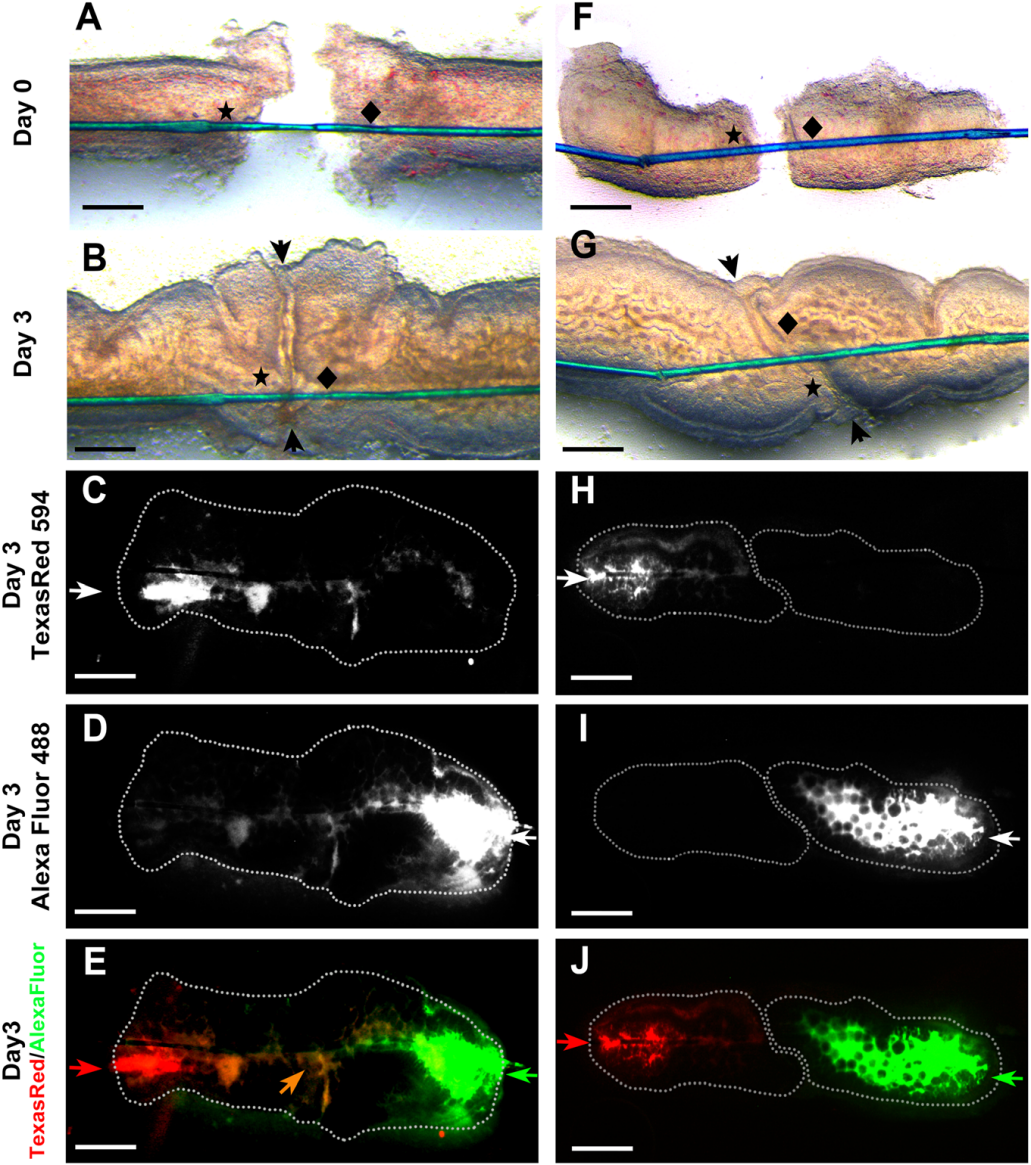
Physical fusion of explanted embryonic jejunum. Images from explants at 0 (A, F) and 3 (other frames) days of culture. (A–E) Pairs of rudiments which fused in culture. (A) Image of rudiments when they were explanted (day 0); note the bridging thread (blue) and the points (indicated by asterisk and diamond) where it enters nearby ends of the rudiments. (B) The same rudiment pair after 3 days of organ culture (day 3); note that the rudiments are well aligned in the border (arrowheads) where they touched. (C–E) Red fluorescent dextran (Texas red) was injected into the left end of the fused organ and green fluorescent dextran (AlexaFluor) injected into the right end; the respective trajectories of the probes are depicted (white) in (C, D) – note that, in each case, the probes flow into the opposite rudiment; (E) merged colour image, with the zone of mixing visible as an orange colour (orange arrow). (F–J) Complementary views of a rudiment pair which failed to form a continuous lumen: after 3 days of culture, although the ends of the explants touched, they were poorly aligned (note the dislocation of asterisk and diamond) and the two dextrans failed to mix and were retained in separate lumens. Bars = (A, B, F, G) 250 µm; (C–E, H–J) 500 µm

By contrast, on day 3 of culture, nine (26%) of 34 rudiment pairs either still had a gap between them or, if their ends touched, the epithelial zones of the two organs were not well aligned (Figure 1F, G). In the latter cases, fluorescent dye was confined within the lumen of the single rudiment into which it had been injected (Figure 1H–J). The average distance between adjacent rudiment pairs on the day they were explanted tended to be lower in well‐aligned (*n* = 25; 181 ± 24 µm, mean ± SEM) vs non‐aligned (*n* = 9; 242 ± 46 µm) pairs (see supporting information, Figure S2) but these values were not significantly different (*p* = 0.21; unpaired Student's *t*‐test).

After 1 day in culture, explanted rudiments began to undergo spontaneous peristalsis, with an average time between the start of contractions of 46 ± 6 s. On day 3 of culture, immunohistochemistry showed that a smooth muscle *α*SMA^+^ zone was present (see supporting information, Figure S1B). Strikingly, in healed rudiment pairs, peristaltic waves were visualized to pass from one rudiment to the other. Sequential still images of a typical wave are shown in Figure 2A–D, with a video of this process included in the supporting information (Video S1). Whole‐mount immunostaining for peripherin revealed a reticular pattern of enteric nerves in rudiments cultured for 3 days. In healed intestines, a subset of peripherin^+^ neurons formed a nexus in the fusion zone between rudiment pairs (Figure 2E–G). This observation would explain how contraction waves might be directed to pass along the fused organ in an uninterrupted manner. Although we do not currently have a method for live imaging of embryonic guts *in vivo*, we recorded (see supporting information, Video S2) similar peristaltic waves in embryonic day 17 jejunum segments that had been explanted in organ culture and then recorded 1 h later. Moreover, a network of peripherin^+^ neurons was detected in the walls of freshly dissected embryonic day 17 jejunum (see supporting information, Figure S3).

**Figure 2:**
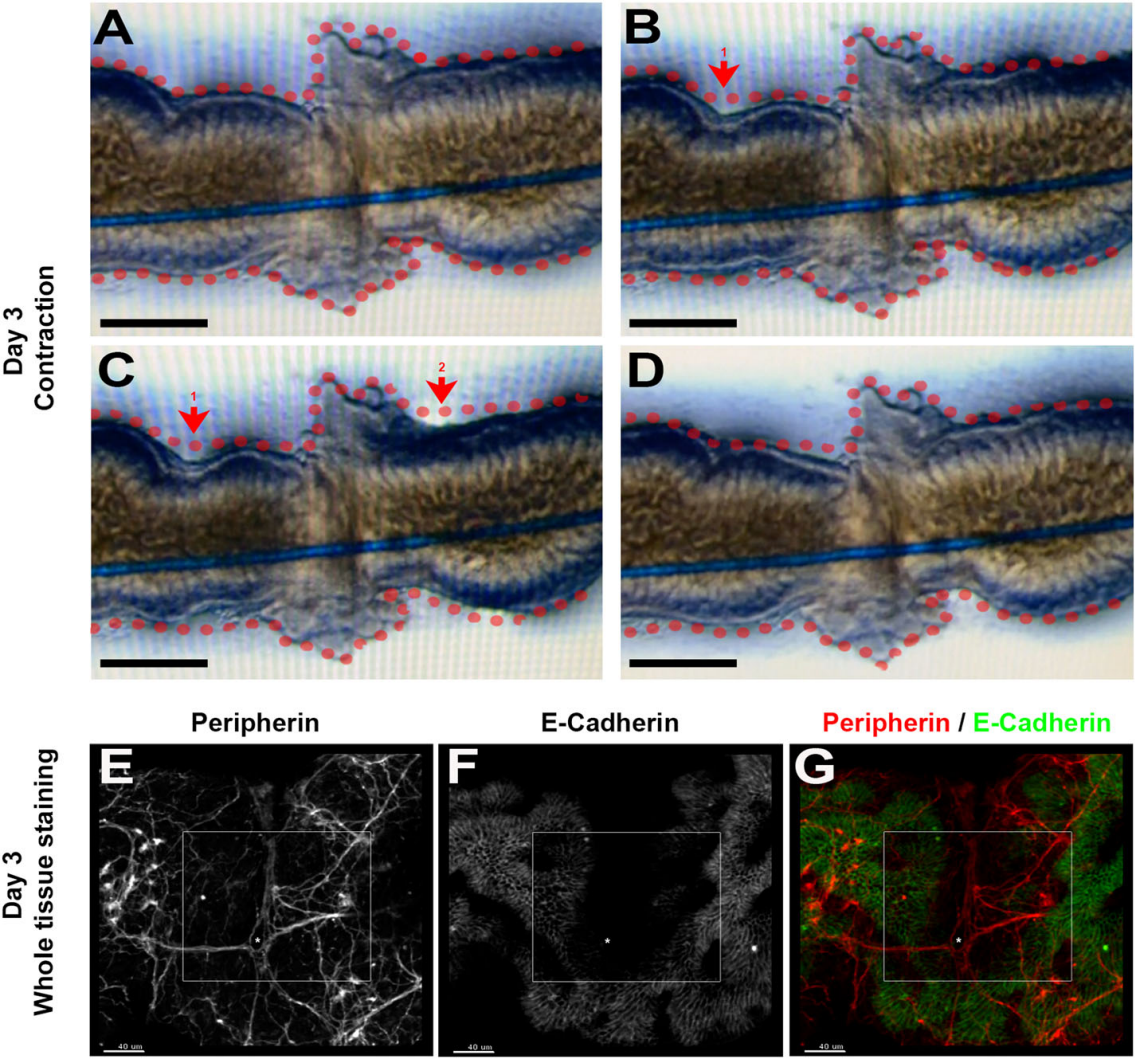
Functional fusion in organ culture. Images from explants at 3 days of culture. (A–D) These four frames are stills, spanning 6 s, taken from Video S•. The fused organ was initially relaxed (starting perimeter is traced by the dotted red lines in A–D). A contraction was spontaneously initiated (red arrow in B) and the wave travelled across the fusion zone, so that both sides of the explant were seen to be contracted (red arrows in C). (D) Finally, the organ relaxed. (E, F) Confocal images showing immunodetection of a peripherin^+^ neural network [white in (E), red in the merged image in (G)] in the wall of a fused explant pair, and the epithelial compartment which is E‐cadherin [white in (F) and green in the merged image in (G)]. The boxed area shows that the neural network traverses the zone where adjacent explants touch; white asterisk, neural nexus. Bars = (A–D) 250 µm; (E–G) 40 µm

Finally, we tested whether addition of 100 ng/ml of R‐spondin 1 affected growth or fusion. The ends of rudiments exposed to this exogenous growth factor displayed sheet‐like extensions by day 3 of culture and these were not found in rudiments grown in basal medium alone (see supporting information, Figure S4, upper frames). Although R‐spondin 1 altered the shapes of the ends of the explants, their lengths at day 3 of culture was not significantly different (unpaired Student's *t*‐test) from explants fed basal medium alone (see supporting information, Figure S4, lower frame). In experiments when rudiments were grown as pairs in the presence of R‐spondin, 14 (67%) of 21 of these pairs were seen to be well aligned and touch after 3 days of culture; this result was not significantly different from that measured in basal medium alone (*p* = 0.76, Fisher's exact test, two‐tailed). The average starting distance between adjacent rudiment pairs in basal medium alone vs pairs in medium supplemented with R‐spondin 1 was not significantly different (*p* = 0.34; unpaired Student's *t*‐test; data not shown).

We believe that insights from experiments to study the healing and growth of intestines in organ culture will ultimately inform the design of novel therapies for human disease. An example of the latter is short bowel syndrome, a potentially fatal condition arising from a variety of developmental and acquired insults, such as congenital atresia and necrotizing enterocolitis (Khalil *et al*., 2012). Surgery to elongate remnant bowel can be undertaken in such patients (Bianchi, 2006), and these treatments can be informed by testing pioneering surgical techniques in large animal models (Cserni *et al*., 2013). The ability to tissue‐engineer guts *ex vivo* is beginning to provide a source of gut tissues that can be used to complement the refashioning of aberrant gut by surgery (Sala *et al*., 2009; Saxena *et al*., 2010). One aspect that has hitherto been little explored is how to elicit optimal functional fusion of the adjacent ends of resected guts.

The current results confirm reports (Abud *et al*., 2005; Quinlan *et al*., 2006) that embryonic murine intestine can be maintained in organ culture, an environment permitting growth and differentiation. Abud *et al*. (2005) used this system to implement signalling through the epidermal growth factor in stimulation of epithelial growth and survival. Quinlan *et al*. (2006) showed that reporter genes could be virally transduced into cultured embryonic intestinal explants. In our experiments, we explored whether paired bowel rudiments could fuse in organ culture to form a single functional unit, as assessed by the formation of a single patent lumen and spontaneous peristaltic waves that spanned the point of fusion of the two rudiments. The key to successful fusion was to span the gap between adjacent rudiments with a thread, which likely provided a bridge along which the nearby ends of adjacent organs could grow and ultimately fuse. In addition, this thread traversed the lengths of adjacent rudiment pairs, keeping them optimally aligned. Not every rudiment pair was observed to functionally fuse and we hypothesized that the addition of R‐spondin 1, an established intestinal growth factor (Kim *et al*., 2005), might increase the frequency of fusion. Although R‐spondin 1 produced outgrowths from the ends of explanted guts, the frequency of fusion was not enhanced, possibly because these extensions had irregular, rather than normal tubular, shapes. Moreover, R‐spondin 1 did not significantly increase the lengths of the explants. In the future we will use the current system as a test bed to assess the effects of other growth factors (Krishnan *et al*., 2011) on the efficacy of gut fusion. We postulate that fusion efficacy could be increased when the added factor confers both an increase in length of the explant together with preservation of the shape of the gut.

### Conflict of interest

The authors declare no conflicts of interest.

## Supporting information

Video S1. Spontaneous peristalsis in healed jejunal neo‐organ
Video S2. Spontaneous peristalsis in an embryonic day 17 jejunum
Figure S1. Histology of embryonic jejunum
Figure S2. Distances between rudiment pairs at the time of being explanted
Figure S3. A neural network in the wall of embryonic day 17 jejunum
Figure S4. Effects of R‐spondin 1 on jejunal explant

Supporting info item

Supporting info item

Supporting info item
