# Supplementary material for: Bridging the gap: functional healing of embryonic small intestine ex vivo
Source: J Tissue Eng Regen Med. 2015 Aug 3;10(2):178–82. doi: 10.1002/term.2073 (PMC4950007; doi:10.1002/term.2073)
Supplement: Supplementary file 3 — Supporting info item [file TERM-10-178-s003.doc]

**Supplemental Material**

**Coletta *et al***

**Bridging the gap: functional healing of embryonic small intestine *ex vivo***

**Supplemental Movie 1. Spontaneous peristalsis in healed jejunal neo-organ.**

Movie of organ depicted in still frames in Figure 2A-B, showing contraction originating in the left part of the fused organ and moving across the zone of healing into the adjacent rudiment. Images of intestinal contraction were observed with a phase-contrast microscope (Leica) and were recorded using 1080p HD video recording camera.

Uploaded on website as file “JTERM Suppl Movie 1.mp4”

**Supplemental Movie 1. Spontaneous peristalsis in an embryonic day 17 jejunum.** Movie of a segment of embryonic day 17 mouse jejunum which had been placed into organ culture one hour before the movie was taken. Note the spontaneous peristalsis, similar to that recorded in embryonic day 14 jejunum cultured for three days (Supplemental Movie 1). Uploaded on website as file “JTERM Suppl Movie 2.mp4”

**
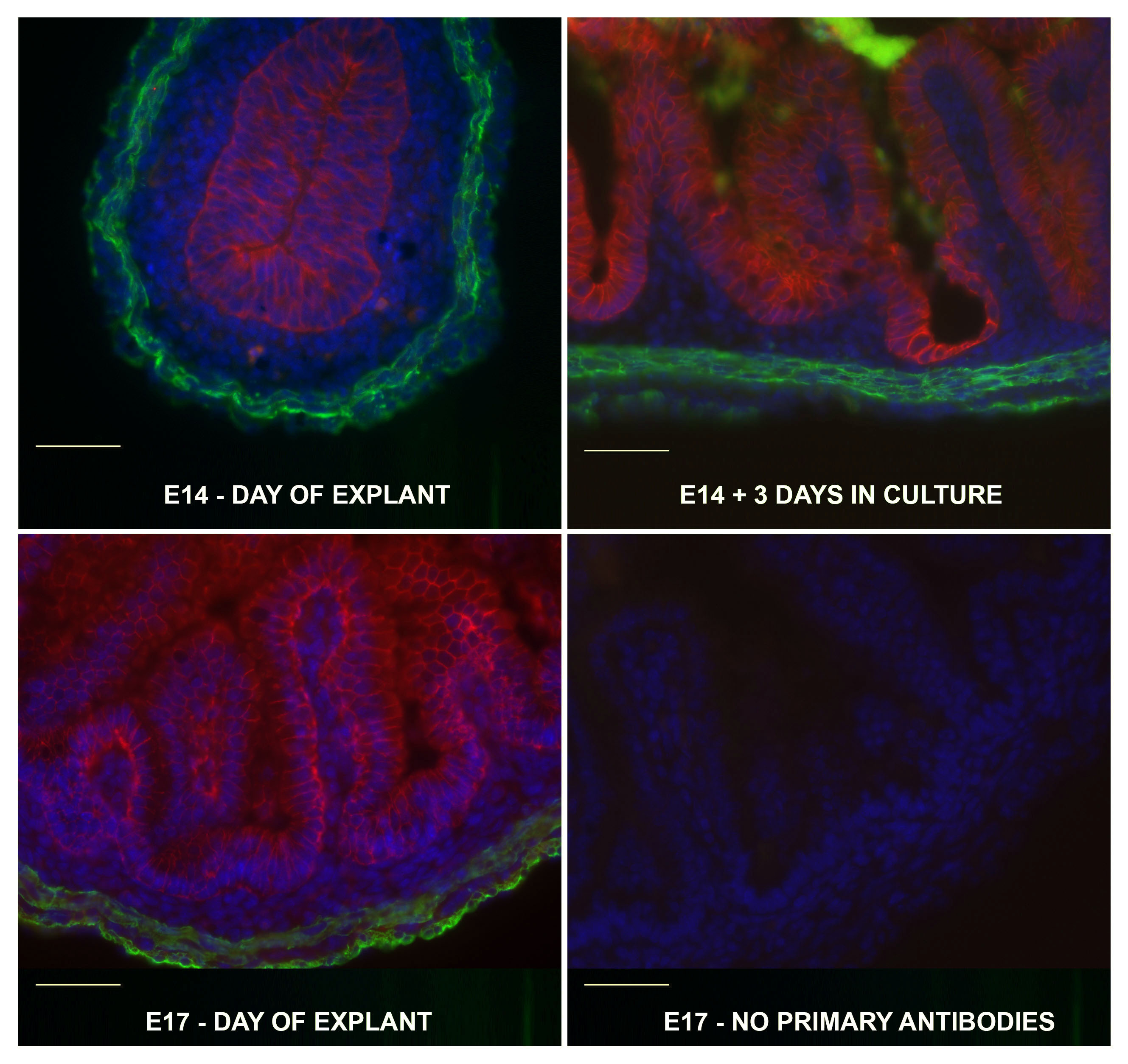
**

**Supplemental Figure 1. Histology of embryonic jejunum double immunostained for αSMA and E-cadherin.** Images are cross sections of intestinal tubes. All nuclei were counterstained with DAPI (blue). αSMA immunostaining appears green and E-cadherin immunostaining appears red. On the day when it was explanted (*E14 – DAY OF EXPLANT*), the rudiment consisted of an E-cadherin^+^ epithelial core which lacked villi; it was surrounded by mesenchyme, the outermost layers of which were αSMA^+^. On day three of culture (*E14 + 3 DAYS IN CULTURE*), rudimentary E-cadherin^+^ villi had formed and smooth muscle was maintained. At this time point, the explant resembled freshly-dissected embryonic day 17 jejunum which also contain villi (*E17 – DAY OF EXPLANT*). The lower right frame (*E17 - NO PRIMARY ANTIBODIES*) depicts a tissue section in which the αSMA and E-cadherin antibodies were omitted. Bars are 50 μm.


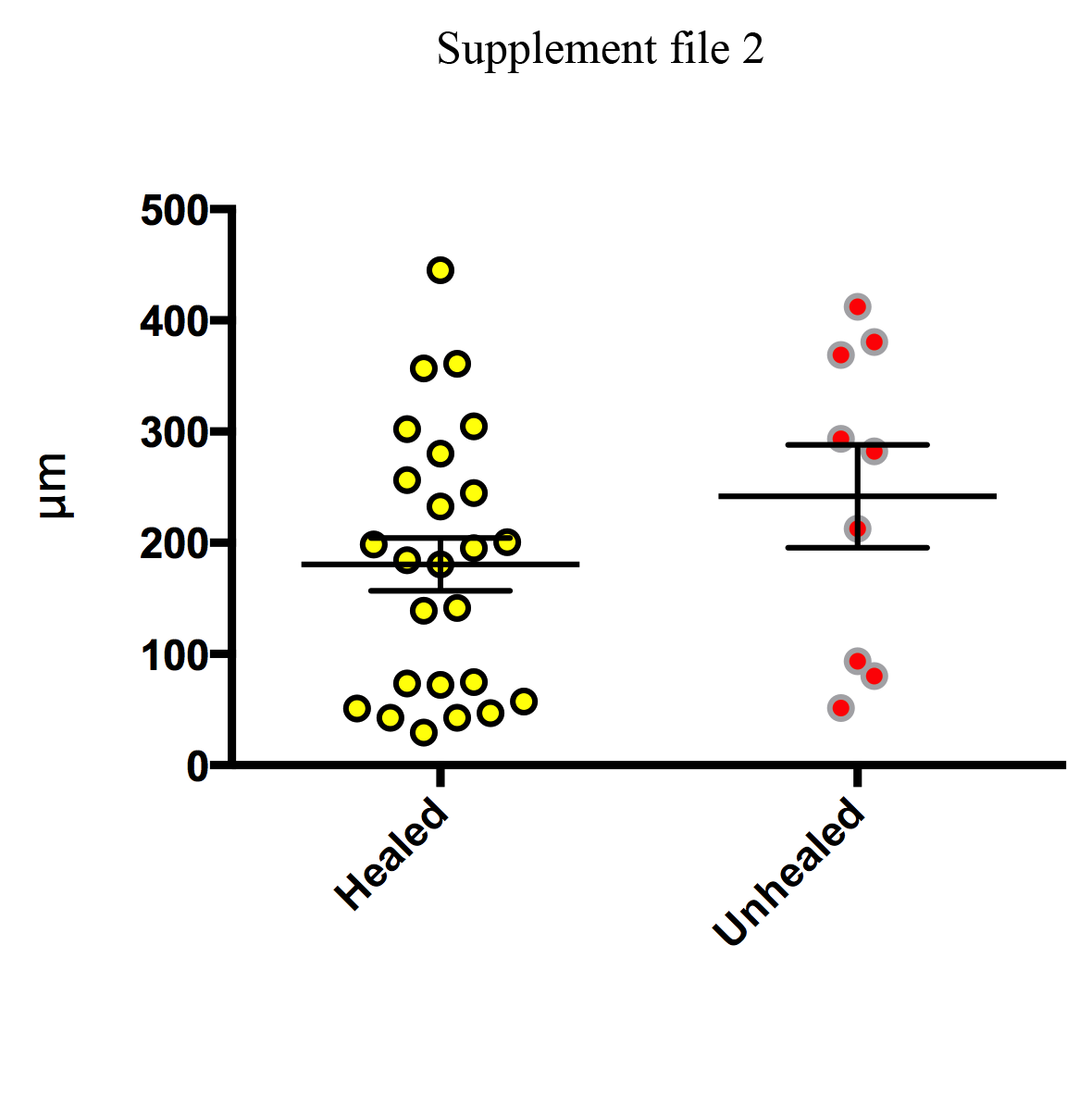


**Supplemental Figure 2. Distances between rudiment pairs at the time of being explanted.** After organ culture in basal medium for three days, a subset of explant pairs healed. There was no significant difference, however, in the starting distances between healed and unhealed pairs.


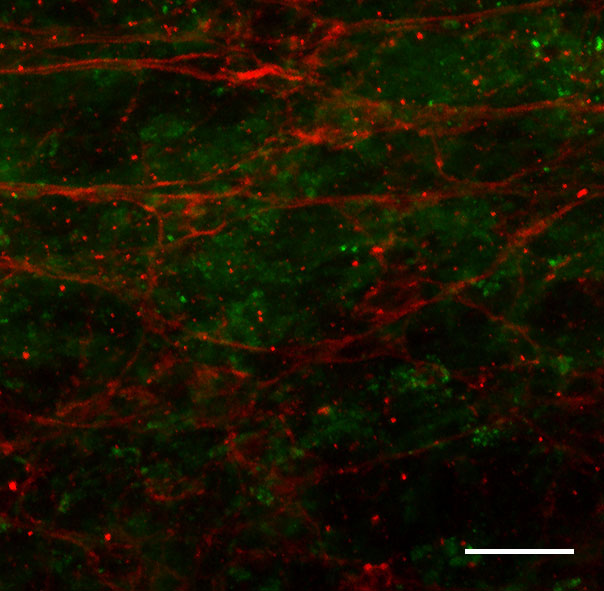


**Supplemental Figure 3. A neural network in the wall of embryonic day 17 jejunum.** Confocal image shows a peripherin^+^ neural network (red) in the wall of a freshly-dissected embryonic day 17 mouse jejunum. Green colour is immunostaining for E-cadherin, which appears relatively faint because only the outermost part of the deeper epithelial layer was imaged here. Bar is 100 μm.


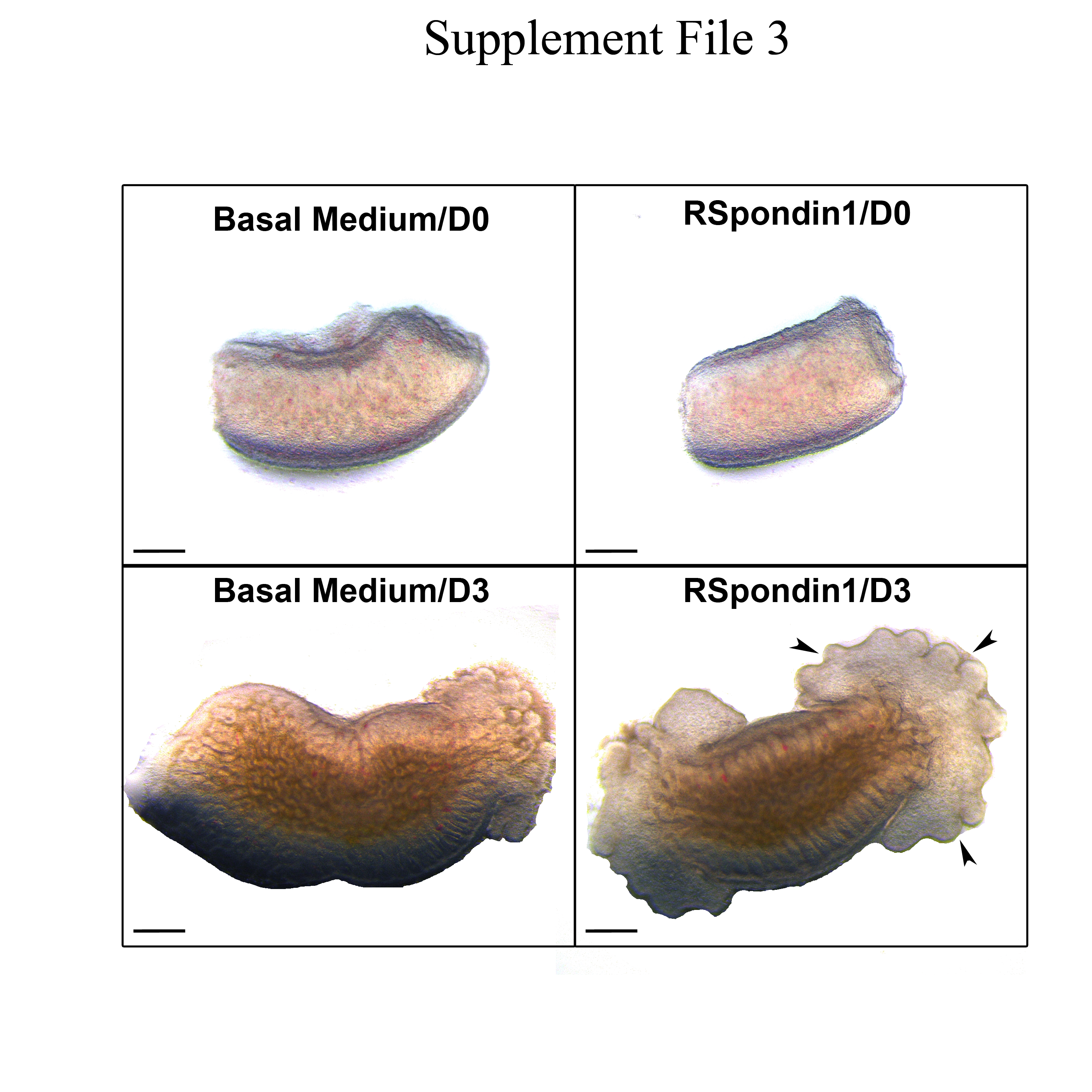


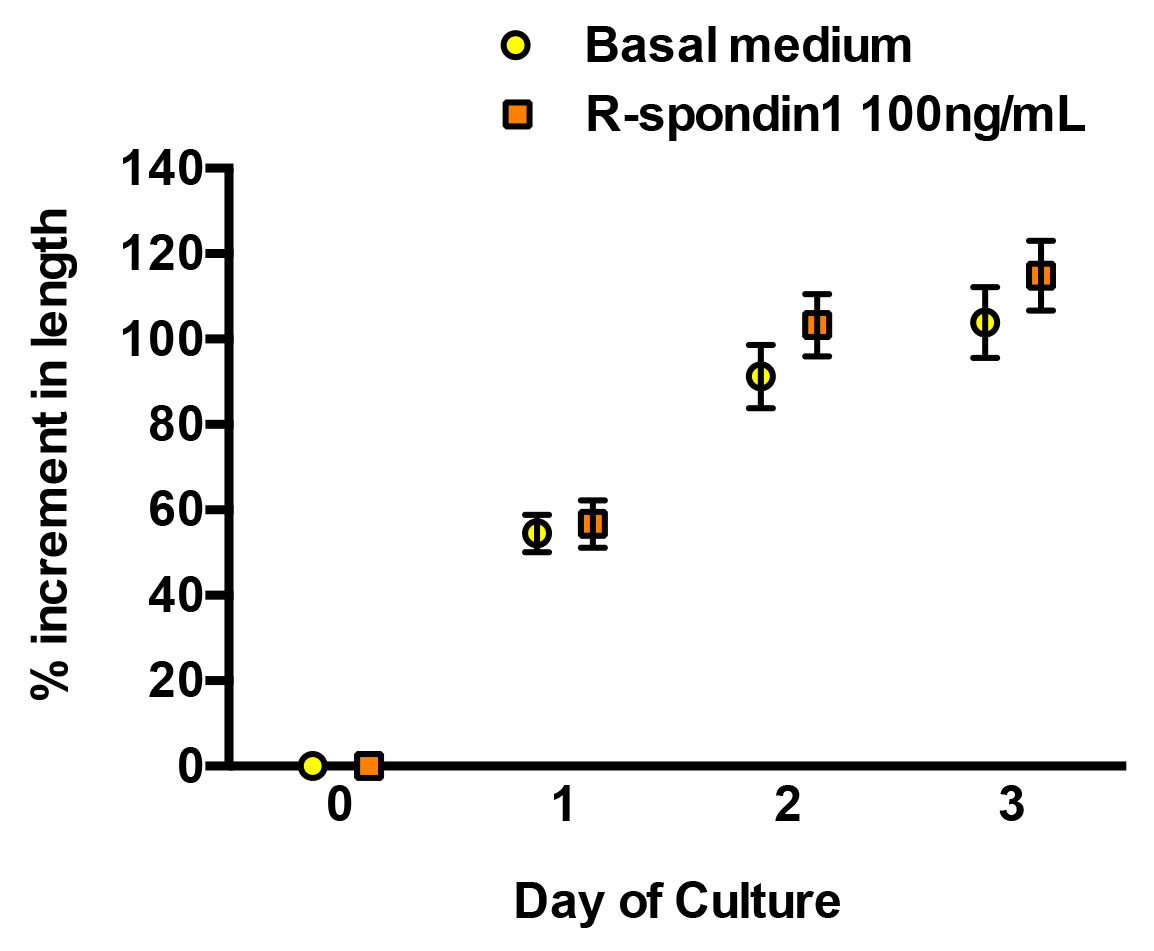


**Supplemental Figure 4. Effects of R-spondin 1.**  **Upper panels.** Jejunal explants were imaged at the day of being explanted (DO) and then after being cultured for three days (D3) in basal media alone or in this media supplemented with R-spondin 1 (100 ng/ml) Note that, in the latter condition, there was exuberant growth of tissue (arrowheads) from the ends of explants. Bars in A-D are 250 μm. **Lower panel.** Although R-spondin 1 altered the shapes of explants, their linear growth was not significantly different (as assessed by unpaired Student’s t-tests) from explants fed basal media alone. Points depict the mean±SEM (n=8 for each condition) % increase in organ lengths over three days in culture.
